# Local Anesthetic Wound Infiltration after Osteosynthesis of Extracapsular Hip Fracture Does Not Reduce Pain or Opioid Requirements: A Randomized, Placebo-Controlled, Double-Blind Clinical Trial in 49 Patients

**DOI:** 10.1155/2018/6398424

**Published:** 2018-11-13

**Authors:** Rune D. Bech, Ole Ovesen, Jens Lauritsen, Claus Emmeluth, Peter Lindholm, Søren Overgaard

**Affiliations:** ^1^Department of Orthopaedics and Traumatology, Odense University Hospital and University of Southern Denmark, Odense, Denmark; ^2^Institute of Public Health, Department Biostatistics, Odense University Hospital and University of Southern Denmark, Odense, Denmark; ^3^Department Anaesthesiology and Intensive Care, Odense University Hospital and University of Southern Denmark, Odense, Denmark

## Abstract

**Background and purpose:**

Local infiltration analgesia (LIA) supports early mobilization after hip and knee arthroplasty. Inspired by this, we studied the effectiveness of wound infiltration with the long acting local anesthetic ropivacaine in an effort to decrease the need for postoperative opioids after osteosynthesis of extracapsular hip fracture.

**Methods:**

Forty-nine patients undergoing osteosynthesis with a sliding hip screw were randomized into two groups in a double-blind study (ClinicalTrials.gov:NCT01119209). The patients received intraoperative infiltration followed by 6 postoperative injections through a wound catheter in eight-hour intervals. 23 patients received ropivacaine and 26 received saline. The intervention period was 2 days, and the observation period was 5 days. In both groups, there were no restrictions on the total daily dose of opioids. Pain was assessed at specific postoperative time points, and the daily opioid usage was registered.

**Results:**

Intraoperative infiltration with 200 mg ropivacaine and postoperative repeated infiltration with 100 mg ropivacaine did not result in statistically significant difference between the groups regarding postoperative opioid consumption or pain.

**Interpretation:**

Ropivacaine as single component in postoperative treatment of pain after hip fracture is not effective. In our setup, wound infiltration with ropivacaine is not statistically significantly better than placebo.

## 1. Introduction

Sufficient treatment of pain is essential for early postoperative rehabilitation after osteosynthesis of hip fractures. The final outcome after hip fracture may be impaired by undertreated pain [1], and conventional methods of pain treatment using paracetamol, systemic opioids, or specific nerve blocks are associated with well-known side effects (sedation, delirium, nausea, and urinary retention) [2, 3]. Local infiltration analgesia (LIA) has proved effective for pain relief after knee arthroplasties [4]. In this paper, we present the data from a randomized clinical trial performed to assess the impact of local anesthetic wound infiltration with ropivacaine for postoperative pain relief after osteosynthesis of extracapsular hip fracture. Our primary purpose was to evaluate if extensive wound infiltration during surgery followed by postoperative repeated injections of extra-articular ropivacaine would reduce postoperative opioid consumption. Secondarily, our aim was to evaluate if wound infiltration in combination with opioid rescue analgesics would cause better pain relief compared to placebo in combination with rescue analgesics.

## 2. Methods

Based on a probable clinical relevant difference of 10 mg of oxycodone per day, a sample size of 13 patients in each study group was calculated (Instant; StatMate, CA). We permitted a type I error of *α* = 0.05 and a type II error of *β* = 0.2. From a pilot study, we estimated SD to 9. To be conservative and to meet the risk of dropouts in this cohort of fragile patients, we decided to include 74 individuals.

**Figure 1:**
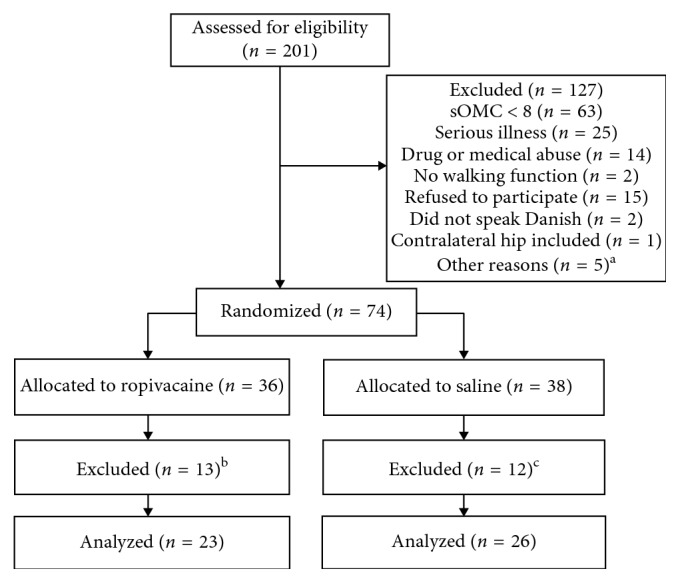
Flowchart of eligible patients. ^a^Five patients were transferred to other hospitals for osteosynthesis before randomization. ^b^Four patients did not receive the allocated intervention. Four patients expected to undergo osteosynthesis with a sliding hip screw were converted to intramedullary nailing. One patient received a femoralis nerve block. One patient was excluded because of significant knee pain caused by the trauma. One patient did not understand the concept of patient-controlled analgesia. Two patients were transferred to surgery in other hospitals. ^c^Four patients did not receive the allocated intervention. One patient received regularly administered opioids and not on demand. One patient accidently removed his catheter. One patient developed delirium before surgery. One patient had insufficient reposition of the fracture and had a re-operation. One patient was transferred to surgery in another hospital. Three patients expected to undergo osteosynthesis with a sliding hip screw were converted to intramedullary nailing.

201 patients with extracapsular fractures (Evans type I-IV trochanteric fractures and basocervical femoral neck fractures) were screened on admission to our department (Figure 1) (Table 1).

Screening was performed prior to surgery by the first author during a period of 3 years. All included participants scored above a specified cutoff point of seven (out of 28) on the inverted version of the Short Orientation-Memory-Concentration Test (SOMC) [5].

The study was approved by the regional ethics committee (Region of Southern Denmark), the Danish Medicines Agency (Copenhagen, Denmark), and was reported to the Danish Data Protection Agency. Registration was done at ClinicalTrials.gov (NCT01119209), and the study was conducted in accordance with the Helsinki Declaration and the principles of Good Clinical Practice. Anonymized research data used to support the findings of this study are available for five years after publication from the corresponding author upon request.

49 patients (ASA I–III) completed the intervention period (Figure 1) after assignment to one of the following two postoperative options: (1) intervention group (23 patients): before wound closure, a bolus installation (75 mL = 200 mg) of ropivacaine was distributed into the tissues surrounding the fracture as described below. This was followed postoperatively by 6 injections (20 mL = 100 mg) of ropivacaine through a catheter placed as described below in 8-hour intervals, until removal of the catheter after 2 days. (2) Placebo group (26 patients): equal amounts of isotonic saline were injected in the same manner as in the intervention group, until removal of the catheter after 2 days. To maintain blinding, the substance for injection was delivered from an external department in anonymous infusion bags prepared according to a computer-generated random code in sequentially numbered sealed envelopes which we received from our statistical consultant. The randomization grouping was revealed when the study population reached end of treatment, and all relevant data were collected, entered, and analyzed in two anonymous groups. Following analysis, group assignment was revealed (placebo and active treatment).

All patients underwent osteosynthesis with a sliding hip screw (Synthes, West Chester, PA 19380, USA) after repositioning of the fracture. Surgery was performed by 35 different surgeons. Standard anesthesia was short acting spinal anesthesia (35 patients) using hyperbaric bupivacaine 0.5% (Marcaine, AstraZeneca Nordic, Sweden), but 14 patients received general anesthesia at the discretion of the anesthesia service because of technical and medical complications. For general anesthesia, we used fentanyl (Haldid, Janssen-Cilag, Belgium) as analgesic component with an expected duration of 30 minutes after intravenous injection. No nerve blocks were performed before or after surgery. Infiltration was done by the surgeon just before skin closure according to an illustrated step by step instruction and supervision by the first author when needed: through the incisional opening and under the guidance of fluoroscopy, 50 mL of the assigned solution was injected into the soft tissues surrounding the fracture and into the anterior and posterior part of the capsule. 25 mL were distributed into the fascia, subcutaneous tissues, and the skin. A multihole ON-Q Soaker Catheter™ (2.5-inch infusion length, I-Flow Corporation, Irvine, CA 92630, USA) was placed along the anterior side of the greater trochanter and connected to a bacterial filter. Wound drains were not used. Dicloxacillin (2 g) was given intravenously before surgery. For thromboprophylaxis, an injection of low molecular weight heparin 5.000 IE was administered subcutaneously for 7 days after surgery. In the case of nausea, metoclopramide 10 mg or ondansetron 4 mg was administered intravenously or orally. All patients were prescribed daily laxative treatment with 10 mg bisacodyl. Treatment of pain was standardized during the whole period of hospitalization. From time of admission to the hospital, all patients received 1 g paracetamol orally 4 times a day supplemented with opioid rescue analgesia as needed. Opioid drugs consisted in immediate-release oxycodone, 5 mg, and there were no restrictions on the frequency or the total daily dose of the opioid drug. During preoperative fast and postoperatively in the recovery ward, rescue medication was provided by the attending nurse as morphine bolus intravenously according to the cutoff values of pain as described in the following section. If patients were unable to rate pain during the recovery phase after general anesthesia, the administration of analgesics were based on the caregivers' subjective estimate of patients' needs.

### 2.1. Study Parameters

The primary outcome measure was consumption of opioid rescue analgesics. We registered the amount of consumed opioid drugs 5 days postoperatively from wound closure. A conversion factor [6] was used to standardize opioid rescue analgesia to an equivalent dose of oxycodone. Pain was assessed on a 5-point Verbal Rating Scale (VRS) with the categories “no pain,” “slight pain,” “moderate pain,” “severe pain,” and “unbearable pain” translated into Danish. Assessments were conducted at rest and by passive straight leg rise to 20 degrees of hip flexion by trained nurses 4 times a day in a fixed pattern. Insufficient analgesia (VRS > “slight pain” at rest or VRS > “moderate pain” by straight leg rise) was relieved by recommending patients to take their oxycodone rescue analgesics. Pain during the daily weight-bearing training sessions was assessed by the attending physiotherapists using the same 5-point VRS. Furthermore, until 5 days postoperatively, we used a 4-point VRS 4 times a day, with the categories “none,” “slight,” “moderate,” and “severe” nausea to assess this well-known side effect.

### 2.2. Statistics

Data entry and statistical analyses were performed with EpiData software (EpiData Association, http://www.epidata.dk) and Stata software (StataCorp LP, Texas, USA). Data were analyzed using the Mann–Whitney *U* test (presented as medians with interquartile ranges) preceded by the Shapiro–Wilk test and Q-Q plots for normality. A *p* value of <0.05 was considered statistically significant. To compare the patients' pain at rest, by passive straight leg rise, and during weight-bearing physiotherapy, we analyzed our data by examining the maximum and medium pain ratings reported at rest and by passive straight leg rise. We also analyzed the number of pain scores that exceeded the predefined limits of tolerable pain (VRS > “slight pain”at rest or VRS > “moderate pain” by straight leg rise) as a measure of sufficient treatment of pain. This stratification of the data provides us with information about the quality of pain relief offered to the 2 groups of patients. Boxplots show interquartile ranges [P_25_–P_75_] with medians highlighted. Lengths of whiskers cover the interval from (P_25_ − 1.5 ∗ interquartile range) to (P_75_ + 1.5 ∗ interquartile range) or the nearest inside value. Outliers from this interval are indicated as circles.

## 3. Results

49 patients completed the study protocol. 23 in the intervention group and 26 in the placebo group (Figure 1). Demographic data were similar between the groups with respect to sex, age, weight, and height, as well as anesthetic technique and days from admission to surgery. Duration of surgery was shorter in the intervention group (Table 2). We observed no infections related to the wound or catheter canal.

### 3.1. Consumption of Opioids

**Figure 2:**
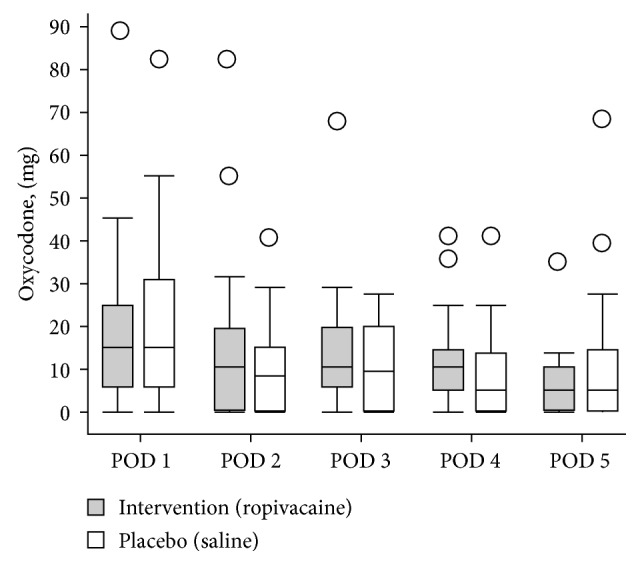
Consumption of oxycodone, *all patients*: (POD 1: *p*=0.9), (POD 2: *p*=0.5), (POD 3: *p*=0.4), (POD 4: *p*=0.3), and (POD 5: *p*=0.7). Postoperative day (POD).

**Figure 3:**
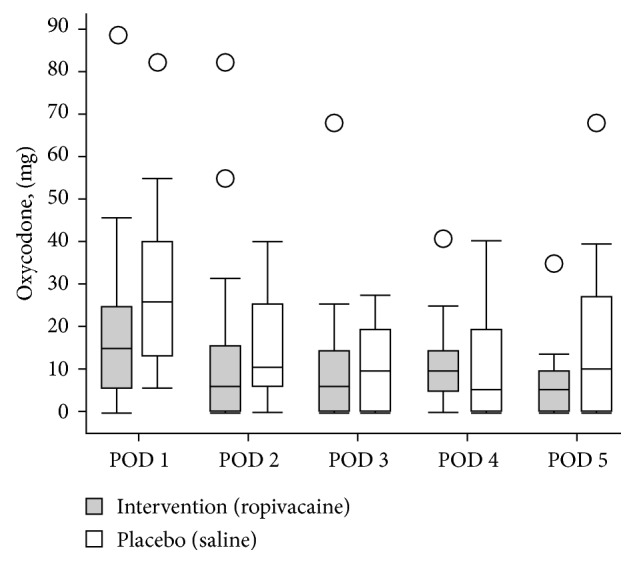
Consumption of oxycodone in the *spinal anesthesia group*: (POD 1: *p*=0.1), (POD 2: *p*=0.4), (POD 3: *p*=0.8), (POD 4: *p*=0.6), and (POD 5: *p*=0.5). Postoperative day (POD).

No statistically significant differences were found in consumption of oxycodone between the 2 groups during the observational period (Figure 2) indicating no effect of the intervention on analgesic requirement. Although not statistically significant, we noticed that patients who received the planned spinal anesthesia and were allocated to ropivacaine infiltration showed a trend toward lower median consumption of rescue analgesia during the intervention period and the following day equivalent to 11 mg (≈2 standard rescue dosages) on the 1st postoperative day (POD 1) and 6 mg (≈1 standard rescue dosage) on the 2nd and 3rd postoperative days (POD 2) and (POD 3), compared to placebo (Figure 3).

### 3.2. Pain Measurements

Table 3 shows pain scores during the intervention period. Pain at rest and at hip flexion was low in both groups without statistically significant difference. We also compared the maximum pain registered per day as a measure of torment in the groups, but no difference was found between the groups, neither at rest nor at hip flexion. Testing for insufficient use of rescue analgesia by comparing the number of pain scores exceeding “slight pain” at rest and “moderate pain” at hip flexion and assessment of pain during weight-bearing physiotherapy showed no difference either. Also on POD 3–5, we found no differences in pain between the groups.

### 3.3. Complications

We did not observe any serious adverse events directly related to the experimental treatment, and there were no statistically significant differences in the occurrence of side effects between the groups (Table 4). The patients' need for antiemetics was similar (metoclopramide *P*=1.0), (ondansetron *P*=0.6) during POD 1–5.

## 4. Discussion

To our knowledge, this is the first randomized clinical trial evaluating the effect of wound infiltration for postoperative pain relief after osteosynthesis of extracapsular hip fractures. In previous studies, we investigated the effect of wound infiltration with ropivacaine in intracapsular hip fractures [7] and after periacetabular osteotomy [8] without substantial effect, but other studies have reported enhanced postoperative analgesia and reduced need for opioid analgesics using the infiltration technique after total knee arthroplasty [9, 10] and total hip arthroplasty [11, 12] which motivated our study. One retrospective study has also attributed the technique to reduced inpatient stay and mortality after hip fracture [13]. Andersen et al. [14] observed reduced need of opioids postoperatively with infiltration intraoperatively followed by top up via catheter. Andersen et al. [11] found reduced pain and a lower requirement for rescue medication from 8 to 96 hours postoperatively using intraoperative periarticular injection followed by top up by means of an intra-articular catheter on day 1, and in a case study of 325 patients, Kerr and Kohan [15] reported acceptable pain scores and no requirements for additional morphine postoperatively in two-thirds of their patients after perioperative infiltration and postoperative top up via catheter.

Traditional nerve blocks require advanced anesthesiological skills, and they involve potential risks of nerve injury. Not least, they might restrict early mobilization due to motor inhibition. The infiltration method has been reported to be safe and simple to perform, and in the view of the fact that hip fracture patients suffer from substantial postoperative pain and that undertreatment of postoperative pain remains a problem [1, 16], we considered the technique to be a potentially attractive complement to the traditional treatment of postoperative pain for these fragile patients. However, with the numbers available, we could not detect a statistically significant difference among the groups. Postoperative pain and opioid consumption was similar in the two groups. During the first 3 days after surgery, we observed decreased opioid consumption in the interventional subgroup receiving spinal analgesia, yet the results are not statistically significant.

There are several possible explanations for the lack of substantial effect. A striking feature is that the pain ratings are generally low if the patients are at rest. This may affect our results since assessment of the impact of our intervention presupposes a certain level of pain which might be restricted by limited mobility alone.

A weakness in our study is that 14 patients were converted from the planned spinal anesthesia to general anesthesia. Obviously, duration of short acting spinal anesthesia may differ and hence, slight differences in pain immediately after surgery might occur. Varying recovery time after general anesthesia, on the other side, necessitates that assessment of pain and administration of analgesics are carried out by caregivers in the recovery ward based on their subjective estimate of patients' needs. Since healthcare workers tend to underestimate the amount of pain that patients experience [17], general anesthesia induces bias to registrations of consumed analgesia in the recovery ward. In our study, the median consumption of oxycodone on POD 1 was statistically significantly lower after general anesthesia (general anesthesia/spinal = 5.7 mg/20 mg, *P*=0.005), which fits with these previous findings. This supports the exclusion of general anesthesia patients when quantifying intake of rescue analgesia (Figure 3). Also, the fact that surgery was performed by thirty-five different surgeons might cause variations in infiltration techniques despite step by step instruction and supervision. Although the infiltration technique is simple, this may lead to more variation in our results.

The patients received 500 mg ropivacaine during the first 24 hours of intervention, and 300 mg during the following 24 hours. The daily dose of ropivacaine administered was less than the maximum dose of 800 mg/day as recommended by the manufacturer (AstraZeneca), and we did not observe adverse effects directly attributable to the injections. We chose the 75 mL volume to ensure sufficient amount for the extensive perioperative infiltration. Likewise, the 20 mL volumes for catheter injections were chosen to aim for a widespread distribution of the solution. In contrast to other investigators [9, 11, 12, 14, 18] we avoided the addition of NSAIDs in order to reduce side effects like decreased bone healing [19], renal toxicity, bleeding, and cardiovascular risk [20]. The risk of these adverse reactions may be restricted during the limited intervention period, but we chose to exercise caution to accommodate with the general fragility of the elderly individuals in the intervention group. Epinephrine was not added because ropivacaine has in itself vasoconstrictive properties [21, 22]. Furthermore, the addition of epinephrine is suspected as the cause of wound necrosis in a previous study [23]. We chose the repeated single shot technique rather than continuous wound instillation, because we expected the single shot technique to increase the likelihood of exceeding a possible threshold of effective concentration in the affected tissues.

Pain was rated using the 5-point Verbal Rating Scale which we have found reliable for assessment of pain after hip fracture in a previous study [24]. Cognitive status was assessed by admission using the six-item SOMC which is validated to test for cognitive impairment [5, 25] since our protocol prescribed a certain cognitive level to ensure that all subjects were able to administer analgesics on demand and to evaluate pain reliably. Using consumption of rescue analgesia as primary parameter to evaluate the effect of wound infiltration presupposes that pain levels are comparable in the 2 groups since it is conceivable that low consumption of rescue analgesia in one of the groups is merely a consequence of insufficient treatment of pain. The pain scores in our study (Table 3) indicate equal and sufficient treatment of pain in both groups, thus we are confident that comparison of rescue analgesic consumption is a reliable method to evaluate the wound infiltration technique used in this study.

The strength of our study is the randomized design with blinding of surgeons, patients, caregivers, and assessors which limits bias by diminishing conceivable differences in outcome caused by other factors than the intervention.

Since our data does not support the practice of wound infiltration with ropivacaine after osteosynthesis of extracapsular hip fracture, we do not use the technique any longer.

## Figures and Tables

**Table 1:** Criteria for Inclusion and exclusion.

Inclusion criteria	Exclusion criteria
Trochanteric fracture or basal cervical fracture	Drug intolerance
Indication for osteosynthesis with a sliding hip screw	Drug or medical abuse
Fracture due to low energy trauma	Pathological fractures
Ability to walk before trauma	Inflammatory arthritis
SOMC (Short Orientation-Memory-Concentration) test ≥ 8 with a possible maximum of 28 points	Patient included in the study with the contralateral hip
Informed consent	

**Table 2:** Patient characteristics, median (range).

	Intervention group (ropivacaine) (*n*=23)	Placebo group (saline) (*n*=26)
Sex (men/women)	7/16	5/21
Age (yr)	83 (50–94)	80 (56–93)
Weight (kg)	56 (50–85)	61 (43–85)
Height (cm)	166.5 (150–185)	165 (152–189)
Anesthesia (GA/spinal)	4/19	10/16
Days from admission to surgery	2 (0–4)	2 (0–5)
Duration of closed reposition and surgery including infiltration and catheter placement (min)	55 (35–115)	81 (43–170)
General anesthesia (GA)		

**Table 3:** Assessments of pain.

	Group		Intervention/placebo *n*^2^	*p*-value
	Intervention^1^ (ropivacaine)	Placebo^1^ (saline)		
Pain (VRS 1–5):				
VRS at rest POD 1	1.5 (1–2)	1.5 (1–2)	11/20	0.9
VRS at rest POD 2	1.5 (1–2)	1 (1–2)	11/20	0.2
VRS, straight leg raise POD 1	3 (2–3)	3 (2–3)	11/20	1.0
VRS, straight leg raise POD 2	2.5 (2–3)	2 (2–3)	11/20	0.5
Max. VRS at rest POD 1	2 (1–3)	2 (1–2)	11/20	0.7
Max. VRS at rest POD 2	2 (1–3)	2 (1–2)	11/20	0.2
Max. VRS, straight leg raise POD 1	3 (3–4)	3 (3–4)	11/20	0.7
Max. VRS, straight leg raise POD 2	3 (2–3)	3 (2–3)	11/20	1.0
Number of pain scores > “slight pain” at rest POD 1	1 (1–2)	2 (1–2)	11/20	0.8
Number of pain scores > “slight pain” at rest POD 2	2 (0–2)	1 (0–2)	11/20	0.5
Number of pain scores > “moderate pain” at straight leg raise POD 1	0 (0–1)	0 (0–1)	11/20	0.7
Number of pain scores > “moderate pain” at straight leg raise POD 2	0 (0–0)	0 (0–0)	11/20	1.0
VRS during daily weight-bearing exercise programme POD 1	3 (3–4)	3 (3–4)	19/20	0.7
VRS during daily weight-bearing exercise programme POD 2	3 (3–3)	3 (3–4)	20/22	0.2

VRS: Verbal Rating Scale; POD: postoperative day. ^1^Values are median (interquartile ranges). ^2^*n*: number of registrations in the two groups.

**Table 4:** Complications and side effects.

	Group		Intervention/placebo (*n*)^2^	*p*-value
	Intervention (ropivacaine)	Placebo (saline)		
Nausea (VRS 1–4):				
VRS at POD 1	1 (1-1)^1^	1 (1-1)^1^	11/20	0.1
VRS at POD 2	1 (1-1)^1^	1 (1-1)^1^	11/20	0.3
VRS at POD 3	1 (1-1)^1^	1 (1-1)^1^	8/16	0.7
VRS at POD 4	1 (1-1)^1^	1 (1-1)^1^	6/9	0.4
VRS at POD 5	1 (1-2)^1^	1 (1-1)^1^	5/6	0.8
Pneumonia (*n*)^2^	2	1	23/26	0.6
Urinary tract infection (*n*)^2^	3	6	23/26	0.5
Other complications (*n*)^2^	5^3^	1^4^	23/26	0.1

VRS: Verbal Rating Scale; POD: postoperative day. ^1^Values are median (interquartile ranges). ^2^*n*: number of registrations in the two groups. ^3^One anaphylactic reaction with pruritus and decrease in blood pressure after administration of dicloxacillin preoperatively. Good response to H_1_ antagonist and hydrocortisone. One case of suspected deep vein thrombosis which was disproved by ultrasonography. One case of diverticular bleeding on POD 7. One case of atrial flutter preoperatively. One case of anemia (Hb 4.8) at admission. ^4^One case of minor cerebral infarction on POD 8.

## Data Availability

The data used to support the findings of this study are available from the corresponding author upon request.
